# Dietary Acid Load Is Positively Associated with the Incidence of Hyperuricemia in Middle-Aged and Older Korean Adults: Findings from the Korean Genome and Epidemiology Study

**DOI:** 10.3390/ijerph181910260

**Published:** 2021-09-29

**Authors:** Dayeon Shin, Kyung Won Lee

**Affiliations:** 1 Department of Food and Nutrition, Inha University, Incheon 22212, Korea; dyshin@inha.ac.kr; 2 Department of Home Economics Education, Korea National University of Education, Cheongju 28173, Korea

**Keywords:** hyperuricemia, uric acid, dietary acid loads, potential renal acid load (PRAL), net endogenous acid production (NEAP)

## Abstract

Hyperuricemia has been associated with a number of chronic diseases, such as type 2 diabetes mellitus, hypertension, and cardiovascular diseases. Dietary acid load plays a key role in regulating uric acid levels. We hypothesized that potential renal acid load (PRAL) and net endogenous acid production (NEAP) score would be positively associated with the incidence of hyperuricemia. Data from the Health Examinees study, a part of the Korean Genome and Epidemiology Study were used. The PRAL and NEAP scores were calculated to evaluate the dietary acid load. Hyperuricemia was defined as follows: >7.0 mg/dL and >6.0 mg/dL of serum uric acid levels in men and women, respectively. Multivariable Cox proportional hazard models were used to estimate hazard ratios (HRs) and 95% confidence intervals (CIs) for the incidence of hyperuricemia. We identified 2500 new cases of hyperuricemia during a mean follow-up of 5.0 years (223,552 person years). The participants in the highest quartiles of the PRAL and NEAP score had 21% (HR: 1.21, 95% CI: 1.07–1.35, *p* for trend <0.0001) and 17% (HR: 1.17, 95% CI: 1.04–1.31, *p* for trend <0.0001) higher risks for hyperuricemia, respectively, than those in the lowest quartiles, after adjusting for covariates. In this prospective cohort study, a higher dietary acid load was positively associated with a higher incidence of hyperuricemia in Korean adults. This suggests that an alkaline diet may be an effective strategy to reduce the future risk of elevated uric acid levels.

## 1. Introduction

Hyperuricemia, an elevated serum level of uric acid, is associated with numerous comorbidities, including renal [1,2] and cardiovascular diseases [2,3], metabolic syndrome [4], hypertension [5,6], diabetes mellitus [7], and obesity [8]. For example, an analysis of the National Health and Nutrition Examination Survey indicated that U.S. adults with serum uric acid levels of ≥10 mg/dL had several comorbidities, such as chronic kidney disease stage ≥2, hypertension, obesity, heart failure, diabetes, myocardial infarction, and stroke [9]. Thus, the presence of hyperuricemia should prompt the investigation of comorbidities [9].

The risk factors for hyperuricemia include reduced urate excretion, endogenous overproduction due to increased levels of purine degradation, or a combination of both [10]. Urate production is increased by endogenous purine production, high cell turnover states (for example, hemodialysis), and decreased excretion due to metabolic acidosis and renal insufficiency [11]. Importantly, the serum uric acid level is affected by a diet rich in purine. All meats, organ meats, and seafood are rich in purine [12], and high consumption of meat and seafood is associated with the development of hyperuricemia [13,14]. Dairy intake has also been associated with decreased uric acid levels via increased uric acid excretion [15].

Researchers have focused on the role of dietary acid load in relation to uric acid levels. A large, representative, population-based study of German adults showed a positive association between dietary acid load and serum uric acid levels [10]. In Japanese women, a low dietary acid load reflected a higher consumption of fruits and vegetables (alkaline diet) than an acidic diet, resulting in a low serum uric acid level via a high excretion of renal uric acid [16]. Therefore, dietary acid load may be suggested as a major modifiable factor that influences serum uric acid levels.

Two indices, the potential renal acid load (PRAL) and net endogenous acid production (NEAP) score, were established to estimate the dietary acid load. The PRAL includes dietary calcium, phosphorus, magnesium, potassium, and protein, whereas the NEAP score evaluates diet acidity based on dietary protein and potassium [17,18]. Highly acid-generating foods, such as animal proteins, increase the net production of nonvolatile acids [19]. When the body pH reaches the lowest point of the normal range owing to this change in the dietary acid load, mild metabolic acidosis occurs [20,21,22].

If the background and knowledge regarding the association between dietary acid load and hyperuricemia can be established, the potential utility of the dietary acid load in preventing hyperuricemia in advance may become crucial. To address these issues, we aimed to estimate the association between PRAL and NEAP scores and the incidence of hyperuricemia in middle-aged and older Korean adults. Furthermore, we assessed the food group intake according to the incidence of hyperuricemia.

## 2. Methods

### 2.1. Data Source and Study Population

**Figure 1:**
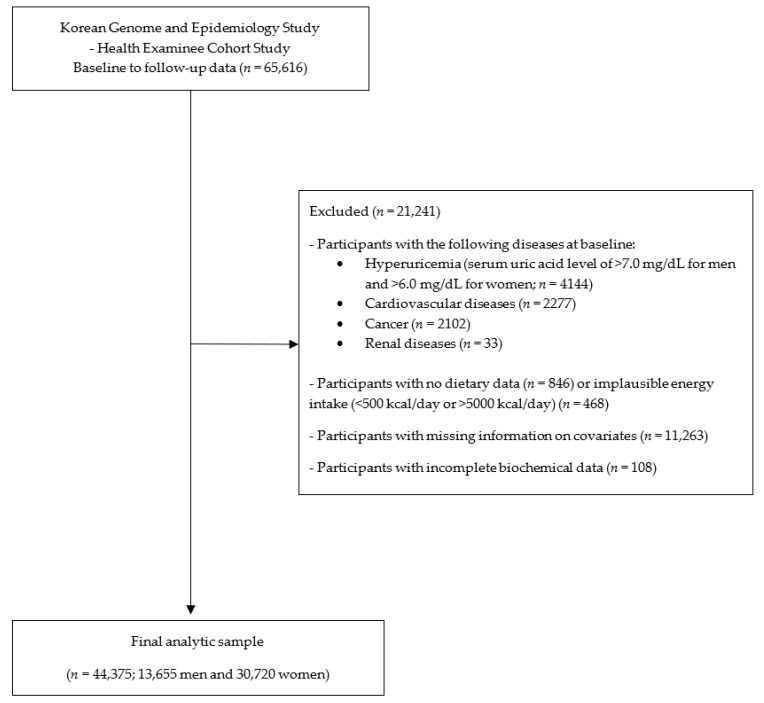
Flowchart of the study population.

The Korean Genome and Epidemiology Study (KoGES) is a population-based prospective cohort study conducted by the Korea National Institute of Health [23]. The main objective of the KoGES was to evaluate the genetic and environmental factors associated with a variety of common diseases [23]. We used the KoGES Health Examinee (HEXA) cohort data, including those of urban residence participants aged ≥40 years. Baseline HEXA data were collected from 2004 to 2013 and the follow-up data from 2012 to 2016. There were 65,616 adults who participated in both baseline and follow-up examinations. We excluded participants who had hyperuricemia (serum uric acid level of >7.0 mg/dL for men and >6.0 mg/dL for women; *n* = 4144), cardiovascular diseases (*n* = 2277), cancer (*n* = 2102), and renal diseases (*n* = 33) at baseline. After the exclusion of participants with no dietary data (*n* = 846) or implausible energy intake (<500 kcal/day or >5000 kcal/day) (*n* = 468), missing information on covariates (*n* = 11,263), and incomplete biochemical data (*n* = 108), a total of 44,375 (13,655 men and 30,720 women) participants were finally included (Figure 1).

### 2.2. Dietary Data, PRAL, and NEAP Score

A validated semi-quantitative food frequency questionnaire (FFQ) with 106 food and beverage items was used to evaluate the dietary intake at baseline [24]. The FFQ consisted of a total of nine frequency items from “never/rarely” to “≥3 times a day” and three portion size options (0.5, 1.0, and 2 standard portion sizes). The frequency consumption of each food and beverage was multiplied by its caloric and nutrient values and then summed for all foods and beverages for each individual. Food and beverage items were aggregated into grains and grain products, rice, vegetables, fruits, meat and meat products, fish and shellfish, and milk and dairy products based on the previous studies [25,26,27].

Using the following formulas, we calculated the PRAL and NEAP scores.

PRAL (mEq/day) = [protein (g/day) × 0.49 + phosphorus (mg/day) × 0.037] − [potassium (mg/day) × 0.0211 − magnesium (mg/day) × 0.0263 − calcium (mg/day) × 0.013] [17].

NEAP (mEq/day) = [protein (g/day) × 54.5/potassium (mEq/day)] − 10.2 [28].

To derive the energy-adjusted dietary acid load from dietary intake, the residual method was used for dietary protein, phosphorous, potassium, magnesium, and calcium intake [29]. Positive scores indicated a highly acidic diet, and negative scores indicated a highly alkaline diet.

### 2.3. Incidence of Hyperuricemia

Hyperuricemia was defined as a serum uric acid level of >7.0 mg/dL in men and >6.0 mg/dL in women, according to previous studies [30,31,32,33]. The endpoint for this analysis was the date of follow-up examination.

### 2.4. Statistical Analyses

Sociodemographic, lifestyle, and dietary factors were expressed as means and standard deviations (SDs) for continuous variables and *n* (%) for categorical variables according to the quartiles of the PRAL and NEAP scores. The chi-square test was used for categorical variables and a general linear regression model was used for continuous variables to examine whether there was a significant difference in each variable according to the quartiles of the PRAL. Three models of multivariable Cox proportional hazard models were used to calculate the hazard ratios (HRs) and 95% confidence intervals (CIs) for hyperuricemia according to the quartiles of the PRAL and NEAP score: model 1 was unadjusted; model 2 was adjusted for age (years, continuous) and sex (men or women); and model 3 was additionally adjusted for educational level (≤elementary school, middle school, high school, or ≥college), smoking status (non-smoker, past smoker, or current smoker), drinking status (non-drinker, past drinker, or current drinker), physical activity (no or yes), body mass index (BMI; kg/m^2^, continuous), and examination site. All statistical analyses were performed using the SAS software (version 9.4; SAS Institute, Inc., Cary, NC, USA). Statistical significance was set at *p* < 0.05.

## 3. Results

In this study, the median PRAL and NEAP score were 4.1 mEq/day and 42.4 mEq/day, respectively. Sociodemographic and lifestyle factors were presented according to the quartiles of the PRAL score. Age, sex, educational level, smoking status, drinking status, and physical activity significantly differed according to the quartiles of the PRAL (all *p* < 0.0001). The individuals in the highest quartile of the PRAL were significantly younger, more likely to be men, had higher than college-level education, were current smokers and drinkers, and were not involved in physical activity as compared to those in the lowest quartile (Table 1).

Table 2 shows the nutrient intake, food group consumption, and biochemical data according to the quartiles of the PRAL. The energy intake, percentage of energy from carbohydrate, fat, total protein, plant protein, and animal protein, and consumption of dietary fiber, phosphorous, potassium, calcium, and magnesium significantly differed according to the quartiles of the PRAL (all *p* < 0.05). The intake of total energy and percentage of energy from fat, total protein, and animal protein were the highest among those in the highest quartile of the PRAL. Meanwhile, the percentage of energy from carbohydrate and plant protein and consumption of dietary fiber, potassium, and calcium were the highest among those in the lowest quartile of the PRAL. The consumption of vegetables, fruits, meat, fish and shellfish, and milk and dairy products significantly differed according to the quartiles of the PRAL (all *p* < 0.05) (Table 2). The intake of meat, fish, and shellfish was the highest among those in the highest quartile of the PRAL, whereas the intake of vegetables, fruits, and milk and dairy products was the lowest among those in the highest quartile of the PRAL. The triglyceride and total cholesterol levels significantly differed according to the quartiles of the PRAL (all *p* < 0.05); high-density lipoprotein-cholesterol level did not differ (*p* = 0.49).

During a mean follow-up of 5.0 years (223,552 person years), 2500 new cases of hyperuricemia were reported. The relationship between the quartiles of the energy-adjusted PRAL and NEAP scores and the incidence of hyperuricemia is presented in Table 3. Those in the highest quartile of the PRAL had a 27% higher risk of hyperuricemia than those in the lowest quartile, after controlling for sex and age (HR: 1.27, 95% CI: 1.14–1.43, *p* for trend <0.0001). Similar findings were found for the NEAP score (HR for the fourth vs. first quartile: 1.24, 95% CI: 1.11–1.39, *p* for trend <0.0001). The associations between dietary acid load and hyperuricemia were slightly weaker in the models when the educational level, smoking status, drinking status, physical activity, BMI, and examination site were additionally adjusted for. Those in the highest quartiles of the PRAL and NEAP scores had 21% (HR: 1.21, 95% CI: 1.07–1.35, *p* for trend <0.0001) and 17% (HR: 1.17, 95% CI: 1.04–1.31, *p* for trend <0.0001) higher risks for hyperuricemia than those in the lowest quartiles, respectively. An increase of 1 SD in the PRAL was associated with a 2% (HR per SD: 1.02, 95% CI: 1.00–1.03) increase in the incidence of hyperuricemia; however, no significant increase was observed with an increase of 1 SD in the NEAP score (HR per SD: 1.12, 95% CI: 0.96–1.31) after controlling for the covariates.

The food group consumption and risk of hyperuricemia are presented in Table 4. In the fully adjusted model, those in the highest tertile for the consumption of grains and grain products, rice, and vegetables had a 12%, 15%, and 10% decrease in the incidence of hyperuricemia compared to than those in the lowest tertile, respectively (HR: 0.88, 95% CI: 0.80–0.97; HR: 0.85, 95% CI: 0.77–0.94; HR: 0.90, 95% CI: 0.81–0.99, respectively). The intake of rice, fruits, meat and meat products, and fish and shellfish did not show significant associations with the incidence of hyperuricemia. We further assessed the relationship between serum uric acid levels and alcohol consumption, especially beer and wine (Table 5). We observed that beer intake was positively associated with serum uric acid levels (*p* < 0.0001), while wine intake was not significantly associated with serum uric acid levels (*p* = 0.99).

## 4. Discussion

In this prospective cohort study, dietary acid load evaluated using the PRAL and NEAP scores was found to be positively associated with the future risk of hyperuricemia in middle-aged and older Korean adults. This association was shown in various models: models unadjusted and adjusted for sex and age and models further adjusted for educational level, smoking status, drinking status, physical activity, BMI, and examination site. Our findings on the association between the PRAL and NEAP scores and the incidence of hyperuricemia are in agreement with previous findings [10,16]. Consuming a highly alkaline diet by increasing fruits and vegetables was associated with increased renal uric acid excretion and decreased serum uric acid levels. Although the underlying mechanisms between the dietary acid load and incidence of hyperuricemia are not fully understood, it has been suggested that uric acid excretion is more favorable in alkaline urine pH than in acidic urine pH [16]. Thus, alkaline urine pH from eating highly alkalizing foods is effective for removing uric acid from the body [34].

We further assessed the relationship between food group intake and the incidence of hyperuricemia. A high intake of grains and grain products, vegetables, and milk and dairy products was inversely associated with the risk of hyperuricemia. In agreement with our findings, a cross-sectional study targeting individuals with overweight/obesity and metabolic syndrome from the PREDIMED-Plus cohort reported that hyperuricemia was negatively associated with a high consumption of dairy products [35]. Moreover, in the Health Professionals Follow-up Study, a high DASH dietary pattern score, characterized by a high consumption of fruits, vegetables, and low-fat dairy products, was associated with a low risk of gout, indicating that it is effective in reducing uric acid levels in individuals with hyperuricemia [36]. Furthermore, the Nutrition and Health Surveys in Taiwan showed that consumption of vegetables, such as carrots and mushrooms, was inversely associated with the incidence of hyperuricemia [37]. The third National Health and Nutrition Examination Survey also reported that those who consumed >2 servings of total dairy foods had significantly lower levels of serum uric acid than those who consumed <0.5 servings [13]. Lactose and galactose are prudent in dairy products, which can trigger the urate transporter/channel and consequently decrease the uric acid level in the plasma [38]. Interestingly, in our study, no significant difference was found between the consumption of fish and shellfish and the risk of hyperuricemia. In contrast, the consumption of seafood was associated with a high prevalence of hyperuricemia in the Chinese population [39,40]. These variations may be attributed to the different levels of purine content in seafood owing to the different cooking methods and preparations, and the fact that they metabolize differently and produce different alterations in uric acid metabolism [15,41,42].

In this study, beer and wine intake were separately analyzed in relation to serum uric acid levels. Although serum uric acid levels increased with beer intake, wine intake did not increase with serum uric acid levels. These findings were consistent with those of previous findings [43,44]. It has been suggested that beer has high purine contents; hence, purines ingested from beer may increase serum uric acid concentration [44]. In contrast, wine has a low amount of purines, and non-alcoholic components such as antioxidants may alleviate the effect of alcohol on the serum uric acid levels [44].

High PRAL and NEAP scores have been associated with hyperuricemia and other adverse outcomes, such as type 2 diabetes mellitus [22], renal stone formation [45], frailty [46], and all-cause and cardiovascular mortality in adults [47]. In a large prospective study, a highly acidic diet in women (PRAL of ≥7 mEq/day and NEAP score of ≥51.3 mEq/day) was associated with the incidence of type 2 diabetes compared to an alkaline diet (HR: 1.56, 95% CI: 1.29–1.90 for the PRAL score; HR: 1.57, 95% CI: 1.30–1.89 for the NEAP score) in a fully adjusted model [22]. A high PRAL score was associated with an increased risk of renal stone formation, especially in those with a low consumption of vegetables [45]. In elderly Japanese women, the positive association between PRAL and NEAP scores and frailty may be partly attributed to the foods and nutrients that are components for calculating these scores [46]. Specifically, the consumption of fruits, vegetables, proteins, and minerals was inversely associated with frailty [46]. Both excess alkalinity and acidity in the diet were associated with a high mortality risk specifically related to cardiovascular disease-related causes [47]. The authors suggested that a diet low in magnesium, potassium, and fiber may induce acidosis, thus yielding nutritional requirements [47,48].

The strength of our study is the prospective cohort study design, with detailed information on dietary and lifestyle factors; thus, we were able to adjust for numerous covariates in our models. We also used validated measures, such as the PRAL and NEAP scores, to estimate the potential acid load of overall diets [17]. A limitation of our study is that the dietary assessment was conducted through self-reported methods, which are prone to causing random and systematic measurement errors [46]. To reduce these errors, we obtained the energy-adjusted PRAL and NEAP score using energy-residual methods [29]. Further, genetic factors were not considered in this study. Three urate transporters, URAT1/SLC22A12 [49], GLUT9/SLC2A9 [50], and ABCG2/BCRP [51], have been identified playing crucial roles in regulating the levels of serum uric acid. Common defects of the ABCG2 exporter have been identified to cause elevated serum uric acid and gout [52,53,54]. *ABCG2* variants have been shown to have stronger effects on the risk of hyperuricemia than major environmental risk factors such as aging, obesity, and heavy drinking [55]. Future studies are warranted to elucidate the relationship between *ABCG2* polymorphisms, dietary factors, and hyperuricemia.

## 5. Conclusions

In conclusion, a higher PRAL and NEAP score was prospectively associated with a higher incidence of hyperuricemia among the middle-aged and older Korean adults in this large prospective cohort study. Future studies are warranted to understand the underlying pathophysiology of hyperuricemia in terms of the amounts and types of purine content in foods and beverages consumed by Koreans, and their bioavailability for purine-to-uric-acid metabolism.

## Figures and Tables

**Table 1:** Characteristics of the study participants at baseline according to the quartiles of the PRAL score.

	Quartile 1 (Lowest)		Quartile 2		Quartile 3		Quartile 4 (Highest)		Total		
	*n*	%	*n*	%	*n*	%	*n*	%	*n*	%	*p* Value
*n*	11,093		11,094		11,094		11,094		44,375		
Median PRAL, mEq/day	−6.6		1.6		6.7		12.8		4.1		
NEAP score, mEq/day	31.1		39.2		45.9		55.6		42.4		
Age, years	53.8	7.6	53.5	7.9	53	8.1	52.4	8.1	53.2	7.9	<0.0001
BMI (kg/m^2^)	23.8	2.7	23.7	2.8	23.8	2.8	23.8	2.9	23.8	2.8	0.41
Sex											
Men	2307	20.8	3165	28.53	3755	33.85	4428	39.91	13655	30.77	<0.0001
Women	8786	79.2	7929	71.47	7339	66.15	6666	60.09	30720	69.23	
Educational level											
≤Elementary school	1761	15.87	1786	16.1	1596	14.39	1328	11.97	6471	14.58	<0.0001
Middle school	1902	17.15	1949	17.57	1790	16.13	1673	15.08	7314	16.48	
High school	4404	39.7	4171	37.6	4283	38.61	4227	38.1	17085	38.5	
≥College	3026	27.28	3188	28.74	3425	30.87	3866	34.85	13505	30.43	
Smoking status											
Non-smoker	9336	84.16	8668	78.13	8172	73.66	7592	68.43	33768	76.1	<0.0001
Past smoker	1115	10.05	1465	13.21	1708	15.4	1947	17.55	6235	14.05	
Current smoker	642	5.79	961	8.66	1214	10.94	1555	14.02	4372	9.85	
Drinking status											
Non-drinker	6771	61.04	6097	54.96	5607	50.54	5058	45.59	23533	53.03	<0.0001
Past drinker	366	3.3	339	3.06	324	2.92	429	3.87	1458	3.29	
Current drinker	3956	35.66	4658	41.99	5163	46.54	5607	50.54	19384	43.68	
Physical activity											
No	4364	39.34	4827	43.51	5105	46.02	5194	46.82	19490	43.92	<0.0001
Yes	6729	60.66	6267	56.49	5989	53.98	5900	53.18	24885	56.08	

The *p* value was obtained using the chi-square test for categorical variables and a general linear model for continuous variables. PRAL: potential renal acid load; NEAP: net endogenous acid production, BMI: body mass index.

**Table 2:** Nutrient and food groups intake, and biochemical data according to the quartiles of the PRAL score.

	PRAL								
	Quartile 1 (*n* = 11,093)		Quartile 2 (*n* = 11,094)		Quartile 3 (*n* = 11,094)		Quartile 4 (*n* = 11,094)		*p* Value
Nutrient intake									
Energy, kcal/day	1721	564	1725	491	1727	487	1740	571	0.008
% Energy from carbohydrate	75.3	6.3	74.3	5.9	73	6.2	69	8.2	<0.0001
% Energy from fat	12	4.9	12.2	4.8	12.9	5	15.2	6.3	<0.0001
% Energy from total protein	12.5	2.6	12.2	2.3	12.4	2.3	13.7	3.1	<0.0001
% Energy from plant protein	7.8	1.3	7.5	1	7.4	0.9	7.3	1.1	<0.0001
% Energy from animal protein	4.7	2.4	4.6	2.3	4.9	2.4	6.5	3.4	<0.0001
Dietary fiber, g/day	17.4	8.8	12.2	5.6	10.4	5.2	9.4	5.3	<0.0001
Phosphorous, mg/day	921.1	375	859.8	315.5	842.7	312.4	885.4	388	<0.0001
Potassium, mg/day	2832	1129.8	2254	774.4	2025.4	734	1907.4	828.6	<0.0001
Calcium, mg/day	571.6	301.6	475.7	237.9	433.4	223	412	241	<0.0001
Magnesium, mg/day	158.1	79.4	123.7	59.5	112.3	56.2	113	63.4	<0.0001
Food groups									
Grains and grain products, g/day	577.5	214.5	662.5	191	691.2	194.1	677.5	223.6	<0.0001
Rice, g/day	516.6	205.9	588.7	179	606.1	177.6	573	191	<0.0001
Vegetables, g/day	431.8	250.8	285.6	135.6	230.6	121	185.9	122	<0.0001
Fruits, g/day	333.4	270.7	196.7	134.4	138	111.8	95.9	91.2	<0.0001
Meat, g/day	32	32.8	38	35.9	45.4	41.8	70.5	73.2	<0.0001
Fish and shellfish, g/day	39.4	36.9	36.4	32.1	38.1	35.3	49.7	54.1	<0.0001
Milk and dairy products, g/day	149.1	170.1	130	146.5	114.7	128.4	99	115.1	<0.0001
Biochemical data									
Triglyceride level (mg/dL) (Reference range: <150 mg/dL)	116.6	74.5	119.7	81.3	122.4	85.7	123.3	86.8	<0.0001
Total cholesterol level (mg/dL) (Reference range: <200 mg/dL)	197.8	35	197.6	34.6	197.2	34.9	196.9	34.9	0.04
HDL-cholesterol level (mg/dL) (Reference range: ≥40 mg/dL for men and ≥50 mg/dL for women)	54.9	12.7	54.7	12.7	54.5	12.9	54.8	13.2	0.49

The *p* value was obtained using a general linear model for continuous variables. PRAL: potential renal acid load, HDL: high-density lipoprotein.

**Table 3:** HRs (95% CIs) for hyperuricemia according to the dietary acid load based on the PRAL and NEAP scores.

	Dietary Acid Load					
	Quartile 1 (Lowest)	Quartile 2	Quartile 3	Quartile 4 (Highest)	*p* for Trend	Per 1 SD Increase
Energy-Adjusted PRAL						
Median, mEq/day	−6.9	1.6	6.7	12.4		4.2
Person years	58,000	55,458	55,273	54,821		223,552
Incident cases (*n*)	539	594	629	738		2500
Rate per 1000 person years	9.3	10.7	11.4	13.5		11.2
	**HR** **(95% CI)**	**HR** **(95% CI)**	**HR** **(95% CI)**	**HR** **(95% CI)**		**HR** **(95% CI)**
Model 1	1.00	1.22	1.29	1.54	<0.0001	1.05
		(1.09–1.37)	(1.15–1.45)	(1.38–1.72)		1.03–1.06)
Model 2	1.00	1.13	1.13	1.27	<0.0001	1.02
		(1.00–1.27)	(1.00–1.27)	(1.14–1.43)		(1.01–1.04)
Model 3	1.00	1.11	1.08	1.21	<0.0001	1.02
		(0.99–1.25)	(0.96–1.21)	(1.07–1.35)		(1.00–1.03)
Energy-Adjusted NEAP Score						
Median, mEq/day	31.2	39.2	45.8	55.5		42.5
Person years	57,650	55,349	55,431	55,122		223,552
Incident cases (n)	528	612	629	731		2500
Rate per 1000 person years	9.2	11.1	11.3	13.3		11.2
	**HR** **(95% CI)**	**HR** **(95% CI)**	**HR** **(95% CI)**	**HR** **(95% CI)**		**HR** **(95% CI)**
Model 1	1.00	1.27	1.29	1.51	<0.0001	1.6
		(1.13–1.43)	(1.15–1.45)	(1.35–1.69)		(1.38–1.84)
Model 2	1.00	1.19	1.13	1.24	<0.0001	1.2
		(1.06–1.34)	(1.01–1.27)	(1.11–1.39)		(1.04–1.40)
Model 3	1.00	1.15	1.06	1.17	<0.0001	1.12
		(1.02–1.29)	(0.95–1.20)	(1.04–1.31)		(0.96–1.31)

Model 1: Unadjusted. Model 2: Adjusted for sex (men or women) and age (years, continuous). Model 3: Adjusted for sex (men or women), age (years, continuous), educational level (≤elementary school, middle school, high school, or ≥college), smoking status (non-smoker, past smoker, or current smoker), drinking status (non-drinker, past drinker, or current drinker), physical activity (yes or no), examination site, and body mass index (kg/m^2^, continuous). The *p* for the trend was calculated using the median value of each dietary acid load parameter in each quartile, treating it as a continuous variable in the model. PRAL: potential renal acid load, NEAP: net endogenous acid production, HR: hazard ratio, CI: confidence interval, SD: standard deviation.

**Table 4:** HRs (95% CIs) for hyperuricemia according to the tertiles of food group consumption.

Food Group Consumption, g/Day	Tertile 1	Tertile 2	Tertile 3
Grains and grain products	1.00	0.87 (0.79–0.97)	0.88 (0.80–0.97)
Rice	1.00	0.88 (0.81–0.96)	0.87 (0.74–1.02)
Vegetables	1.00	0.96 (0.87–1.06)	0.85 (0.77–0.94)
Fruits	1.00	1.07 (0.97–1.18)	1.01 (0.91–1.12)
Meat and meat products	1.00	0.95 (0.86–1.06)	0.98 (0.88–1.08)
Fish and shellfish	1.00	0.96 (0.87–1.06)	0.96 (0.87–1.06)
Milk and dairy products	1.00	1.00 (0.91–1.09)	0.90 (0.81–0.99)

Adjusted for sex (men or women), age (years, continuous), educational level (≤ elementary school, middle school, high school, or ≥ college), smoking status (non-smoker, past smoker, or current smoker), drinking status (non-drinker, past drinker, or current drinker), physical activity (yes or no), examination site, and body mass index (kg/m^2^, continuous). HR: hazard ratio, CI: confidence interval.

**Table 5:** Association between alcohol consumption and serum uric acid levels.

Alcohol Consumption, g/Day	Βeta ^1^	Standard Error	*p* Value
Beer intake	0.0016	0.0003	<0.0001
Wine intake	0.00001	0.0009	0.99

^1^ Adjusted for sex (men or women), age (years, continuous), educational level (≤elementary school, middle school, high school, or ≥college), smoking status (non-smoker, past smoker, or current smoker), drinking status (non-drinker, past drinker, or current drinker), physical activity (yes or no), examination site, and body mass index (kg/m^2^, continuous).

## Data Availability

Data underlying the results of our study are not publicly available due to the KoGES data policy. Data are available from the Division of Genetic Epidemiology and Health Index, NIH, Korea Centers for Disease Control and Prevention (contact via Mi-Jin Cho at whalwls0227@korea.kr) for researchers who meet the criteria for access to confidential data.
